# A comparison of two short-term intensive physical activity interventions: methodological considerations

**DOI:** 10.1186/1479-5868-8-133

**Published:** 2011-12-05

**Authors:** Lynda H Norton, Kevin I Norton, Nicole Lewis, James Dollman

**Affiliations:** 1 School of Medicine, Flinders University, Bedford Park, South Australia, 5042, Australia; 2 School of Health Sciences, University of South Australia, City East Campus, Adelaide, South Australia, 5000, Australia

**Keywords:** physical activity theory, physical activity interventions, adherence, compliance, pedometer

## Abstract

**Background:**

Increases in chronic illness due to sedentary lifestyles and poor metabolic fitness have led to numerous intervention strategies to promote physical activity (PA). This paper describes the methodological strategies of two short-term PA interventions. Outcome measures reported are PA adherence and compliance rates during the intervention and at 3, 6 and 12-month follow-up.

**Methods:**

The 40-day interventions were: a pedometer-based walking program (n = 251) and a group-based intensive program (n = 148). There was also an active control group (n = 135). Intervention subjects were prescribed PA each day and required to record all activity sessions (pedometer steps or energy expenditure from heart rate monitors).

**Results:**

Compliance (≥ 150 min/wk PA) was highest post-intervention (81.1% and 64.5% for the group and pedometer subjects, respectively) and then progressively decreased across the 12-month follow-up period (final compliance rates were 53.5% and 46.6%, respectively) although they remained significantly higher than pre-intervention rates (zero %). There was significantly higher adherence to 6 months (75.0% and 64.9%), and compliance to 3 months (64.9% and 51.0%), for group versus pedometer subjects. The active control group maintained the highest adherence and compliance rates across the study.

**Conclusions:**

The group-based program resulted in higher adherence and compliance rates post-intervention although both types of interventions showed long-term effectiveness to increase activity patterns.

## Background

Rapid and pervasive technological developments of the 20th century have influenced the way humans spend their time [1,2]. These inventions are typically labour-saving devices that reduce energy expenditure [3]. At the same time many nations have recorded rapid rises in the prevalence of overweight and obesity, and concomitant increases in chronic illness such as diabetes and cardiovascular disease [4,5]. Declining levels of physical activity (PA) and the resultant poor metabolic fitness have been found to be important components in the aetiology of these chronic illnesses [6]. Therefore, intervention strategies to promote PA and to quantify the impact on health outcomes have become public health priorities [7].

An enormous variety of interventions have been conducted around the world. Many have been summarized in systematic reviews and meta-analyses [8-11]. Overall, there is considerable heterogeneity in the strategies used to increase PA. These strategies can be categorized to help understand the effectiveness of intervention types to change behaviours. The CDC, for example, conducted a systematic review of 94 PA interventions and identified several strategies they 'recommended' or 'strongly recommended' to increase activity levels [9]. They grouped these into three broad categories - environmental and policy-based, information-based, and behavioural and social approaches, and found successful outcomes came from strategies within each of the intervention approaches.

Other studies have also examined various types of interventions to help identify the strategies most likely to be successful [8,10,12-16]. The findings show interventions vary widely in both methodologies and, when described, in the theoretical underpinnings.

There are literally hundreds of possible design features reported which make it problematic to disentangle the specific elements that are important for successful change. A recent Cochrane review found some PA interventions were moderately effective, but there was a need to establish which methods worked best in the long term (including their composition) and among different types of people [8].

Understanding the theoretical basis of interventions is also important in order to tease out the contributions of a range of mediating variables, subject characteristics and other mechanisms for changing behaviours. There are three predominant theoretical approaches in PA interventions: (1) information-based approaches, such as the health belief model, under the expectation that, once educated, participants will make healthier choices, [17] (2) approaches that are tailored to a specific stage of change of the subjects such as the transtheoretical model, [18] and (3) broader socio-ecological approaches that target a combination of individual, social and environmental strategies [19]. Overall, it is important to link the theoretical approaches to PA interventions and/or mediators of behavioural change and the resultant outcomes, particularly using variables such as compliance over the longer term [20].

Despite the enormous global effort to promote PA there still remains a high proportion of adults who fail to meet current international PA guidelines for optimal health benefits [21-23]. Furthermore, there is a pressing need for information on new intervention strategies particularly compared to those strategies that have already been identified as showing varying degrees of success.

This paper details the theoretical rationale and design strategy for two short-term intensive interventions (40-DAY PA study). One intervention arm is broadly based on a health-belief model while the other takes a more socio-ecological approach. PA adherence and compliance rates, and a range of health-related measures were tracked during the interventions and at 3, 6 and 12-month post-intervention. The study adds to the literature because it quantifies in detail physical activity patterns within each of two large intervention cohorts including intensity, type, duration and frequency of exercise habits. It facilitates a comparison of two intervention approaches to effect PA and sedentary behaviours over the longer-term and how these are related to both fitness and health-related changes.

## Methods

The 40-DAY PA study was a randomised controlled intervention trial designed to increase PA levels of insufficiently active adults. The study involved two intervention arms and an active control group. The intervention subjects were randomised to one of two 40-day activity programs and subjects were followed for 12 months post intervention. Outcome measures included PA patterns and health and fitness-related parameters (reported in a separate paper).

Two types of intervention were used: (1) a limited contact, information-oriented, pedometer-based strategy that was based on the health-belief model, and (2) an intensive, structured, group-based strategy using a multi-layered socio-ecological approach.

### Subjects

The University ethics committee approved this study and all subjects gave informed written consent. A total of 553 subjects aged 18-60 yr enrolled using the following selection criteria:

• 'insufficiently active' according to the Active Australia Survey (AAS) criteria (< 150 min of weighted PA per week) to be part of the intervention arms; [24]

• willing to either (a) wear a pedometer daily for the duration of the 40-day intervention or (b) participate in the 40-day group PA program, or (c) act as controls if they were regularly sufficiently active (averaged ≥ 150 minutes of weighted PA/week for at least the past 12-months)

• satisfy the pre-exercise screening guidelines using Sports Medicine Australia's screening system (http://sma.org.au/wp-content/uploads/2009/05/new_pre_screening.pdf)

### Recruitment

**Figure 1:**
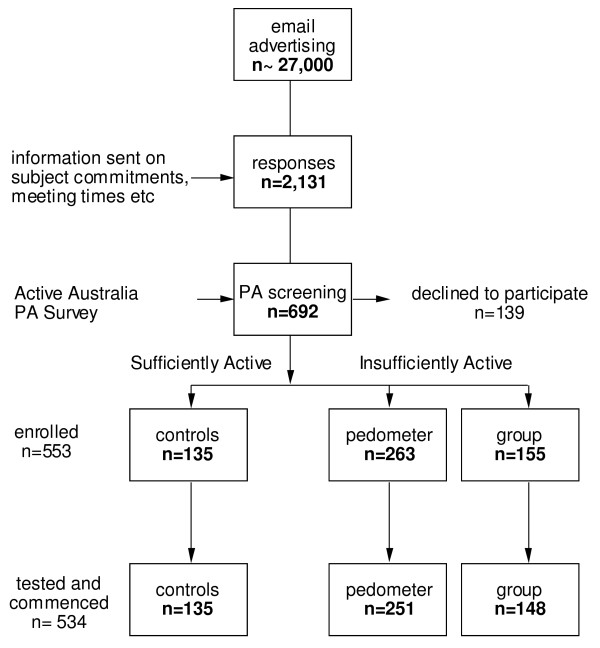
**Flow chart of subject recruitment and selection**.

The sequence of events leading to the recruitment of subjects is illustrated in Figure 1. Recruitment followed email advertising sent throughout a university, tertiary hospital and several government departments. Following an initial email/phone enquiry 2,131 respondents were sent detailed information. Interested participants attended the Exercise Research Laboratory at the University. At the first laboratory session participants completed a PA questionnaire covering the previous week [24]. Total PA time was calculated by adding the time spent in walking and moderate activity plus twice the vigorous activity time (not including gardening and housework). Individuals who had < 150 min of weighted PA per week were invited to undertake the 40-DAY PA intervention. Those who regularly achieved ≥ 150 min/wk over the previous 12-months were invited to participate as active controls. Subjects undertook a formal laboratory orientation to the testing protocols and a second laboratory visit was scheduled for pre-exercise screening, and health and fitness assessments.

### Pre-exercise screening

Pre-exercise screening involved a series of health-related questions and physiological tests to determine whether the subject was recommended for medical clearance before beginning the 40-DAY PA intervention. Briefly, via questionnaire, stage 1 identified people with either signs or symptoms of, or established, disease who were advised to seek medical clearance before beginning PA. Subjects required medical clearance at stage 2 if they had extreme, or multiple, cardiovascular, metabolic or respiratory system risk factors.

### Randomisation

Subjects were randomly assigned to either the group (n = 155) or pedometer (n = 157) intervention arm using computer-generated numbers. Allocation concealment was achieved pre-intervention by having the randomisation process conducted after health and fitness testing. It was not possible to conceal the subject's intervention group during the intervention given the nature of the PA. There were an additional 96 subjects who could not attend the group intervention classes (for various reasons) but were otherwise willing to participate in the study. There were no differences in the age, gender or physical activity patterns between the randomised versus non-randomised subjects either pre or post intervention.

### Intervention theory

A number of theoretical components were incorporated in this study for both intervention arms. The primary intra-personal components addressed were self-efficacy (subjects were taught goal-setting techniques and self-management strategies [25]), and outcome expectancy (eg, the benefits of PA such as improved fitness indicated by lower heart rates and feeling more energetic). Additionally, skill development in a wide range of activities such as yoga, core stability, boxing, kayaking, resistance training, aerobic exercise, team sports [17] was incorporated into the group intervention.

Inter-personal and cultural factors were incorporated into both programs, including strategies to make exercise more enjoyable using individual challenges and motivational music [25], and social support where the program involved strategies to elicit support for PA from family members, friends or work colleagues [26].

Table 1 outlines group intervention strategies to enhance the inter-personal components. These included role-modelling based on subjects participating in group-led PA sessions three times/wk providing them with exposure to a variety of activities under the supervision of experienced exercise leaders [8], and promoting and encouraging PA in a variety of environments. This involved the group-led sessions being conducted in a range of settings utilising community spaces, parks, beaches, walking trails as well as a university gym and sports field [17,27].

**Table 1:** Methodological components of both intervention arms.

Methodological components	
**Group**	**Pedometer**

• intra-personal focus on self-efficacy	• intra-personal focus on self-efficacy
face-to-face group sessions 3x/wk	electronic communication 1x/wk
practical PA skills	
	
• outcome expectancy of health and fitness benefits	• outcome expectancy of health and fitness benefits
	
• inter-personal and cultural factors	• inter-personal and cultural factors
encourage social support	encourage social support
individual challenges	
motivational strategies	motivational strategies
role-modelling PA behaviours	
varying exercise environments and activities	
	
• PA monitoring	• PA monitoring
HR monitors and diaries	pedometers and diaries
weekly downloads of daily HR records	diaries collated post-intervention
	
• goal setting	• goal setting
daily energy expenditure and intensity levels	daily step counts
	
• identifying barriers and enablers	• identifying barriers and enablers
reinforced individually in face-to-face group sessions	reinforced weekly in generic emails
	
• health and fitness testing	• health and fitness testing

PA = physical activity

Other behaviour modification strategies used in this study involved PA monitoring using either pedometers and diaries (pedometer subjects), or heart rate monitors and diaries (group-based subjects). This introduced elements of expectation and personal attention in the knowledge the sessions were checked for compliance [28]. Goal setting was also taught using daily step counts for the pedometer subjects and daily energy expenditure quantified by the heart rate monitors for the group subjects [29]. Barriers to PA were also identified and approaches to help overcome these were reinforced [30]. Specific individual strategies for increasing PA were also identified and promoted every week (eg, incorporating some active transport in getting to and from work, walking at lunch-time, setting the alarm clock 30 min earlier, preparing workout clothes the night before or assembling inspirational music playlists).

Six intervention groups were recruited throughout the year to encompass a range of seasons. Each group participated in one 40-day PA program. Morning and early-evening sessions were offered to the group subjects in April (autumn), July (winter) and September (spring). The pedometer interventions were run at the same time periods as the group interventions.

Subjects in both intervention groups attended an education session prior to the intervention commencing. They received information on the health benefits of regular PA, national PA recommendations [31], 10,000 steps per day guideline and suggestions on how to increase PA in day-to-day life. Instructions on using pedometers and heart rate monitors were given and written instructions were provided in the diary. Pedometer subjects were required to complete their diary daily with information on step counts achieved, type of activity and session duration if applicable, and other information about their exercise, for example, injury, illness, holidays. Group-based subjects entered information on activity type, duration, energy expenditure and heart rate details from the HR monitor, rating of perceived exertion (RPE) for each session, as well as additional information as per pedometer subjects. Given that subjects had initial low activity levels both interventions started conservatively and progressed in intensity and/or volume.

### Pedometer intervention

Pedometer subjects were equipped with a pedometer (Yamax Digiwalker SW-700) for the 40-DAY PA program. Pedometer subjects were emailed walking maps and approximate step counts throughout the local regions to encourage variety and to help set challenges. In the first week of the intervention subjects were instructed to achieve at least 5,000 steps/day. The step count was gradually increased by 1,000 steps/wk to 10,000 by week six. A weekly email was sent to pedometer subjects outlining the step count goal for the week and tips to increase walking activity. It is a methodology used in numerous interventions and summaries of step increases show changes are typically within the range 1,500-2,500 steps per day following similar interventions [32,33]. The pedometer-based intervention in this study is therefore considered a 'usual treatment' [34].

### Group intervention

The group intervention combined elements that have been shown to be important for long-term behavioural change. The program was based primarily on self-efficacy theory proposing that confidence in one's ability to perform activity is strongly related to actually performing that behaviour [17]. Furthermore, the group intervention provided opportunities to enhance social networks and group cohesiveness which have been shown to help sustain participation [35], particularly given the commonality of the 40-DAY PA task-oriented objective.

The intervention educated and motivated with a focus on energy expenditure through activity augmented by the immediacy and security of heart rate monitoring (Polar S610 worn during physical activity sessions greater than 10 min). Individual day (self administered) activities were included and these were also monitored via the heart rate recordings and downloaded each week.

The group-based intervention was designed to promote links between the workplace and the community to allow participants to experience new ways to be active [27]. The requirement for participants to be active on individual days allowed them to incorporate home-based PA while having regular access to instructors to assist in goal setting and PA planning.

The intervention was designed to decrease potential for boredom and residual soreness in previously insufficiently active subjects by regularly rotating muscle groups and body areas used in activities. The group intervention involved a range of progressively scheduled activities including body awareness routines, walking, core stability/flexibility sessions, aerobic circuits, team-building challenges, and modified sports and games.

**Figure 2:**
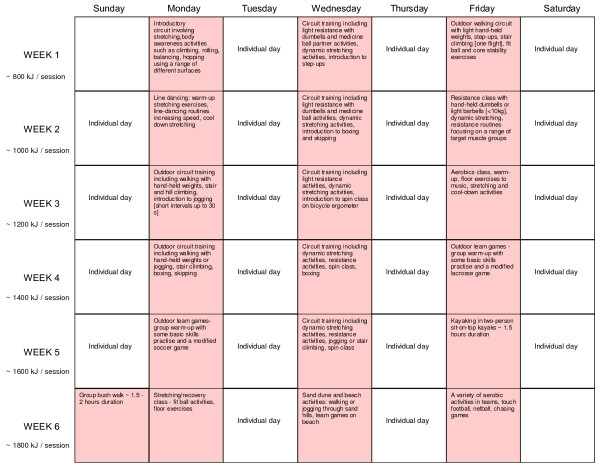
**40-DAY PA itinerary for the group intervention arm showing the 40 consecutive days of activity**.

The fitness instructors provided leadership, instruction, feedback and guidance during the critical early phase of beginning new activities when many people drop out of PA programs [36]. Group intervention subjects attended instructor-led activities three times/wk (Mon, Wed, Fri). Subjects participated in activities of their own choice on alternate days but were required to complete at least 30 min of activity every day for 40 days while wearing their HR monitors. Researchers downloaded HR monitors weekly to record exercise duration, %HRmax and energy expenditure (EE, in kJ). Group sessions were designed to expend approximately 800 kJ in the first week and to increase by about 200 kJ per session in each subsequent week. Figure 2 shows the activities and the progressive increase in EE during the intervention. This was primarily through increased exercise intensity since session times were relatively constant across the program.

All sessions included a 10 min warm-up and a 10 min cool-down/stretching period. Most sessions lasted 60 min and the core involved subjects working at about 60-80% of estimated HRmax. Session intensity was determined on the basis of average HR measurement using the entire session. HR monitors were individually programmed according to the manufacturer's recommendations [37].

### Controls

The controls were physically active beyond the recommended 150 min/wk of moderate activity. The rationale for using active controls was primarily based on the knowledge that being insufficiently active is among the leading causes of premature death and disability [4]. In long-term interventions it is ethically questionable to ask subjects to maintain a level of activity known to be poor for their health [38]. Allocation to a proven standard of harm-minimising behaviour (underpinned by many large-scale studies) is used in pharmacology and medicine and should also be encouraged in physical activity promotion. The active controls therefore represent one form of control in the present study along with the pedometer arm. Randomising insufficiently active subjects to a maintenance control group also introduces the prospect that some participants begin exercise programs of their own (that they may not disclose) from a low base resulting in significant health outcomes making them an unstable reference [15].

### Statistical analysis

Statistical analysis was performed using SPSS software. Analysis of variance (ANOVA) and t-tests were used for between-group comparisons. Non-parametric analyses such as Kruskal-Wallis were used for variables such as skewed PA and sedentary behaviour data and Chi square to determine patterns of adherence and compliance. Adherence was defined as the continuation of the subjects in the study and quantified as the number of subjects returning for laboratory testing. Compliance was calculated in two ways - both using intention to treat (ITT) analysis: (1) during the intervention it involved achieving the prescribed daily activity, either the step count for the pedometer subjects or a minimum of 30 min of recorded activity for the group subjects specifically for week-by-week within-group analysis, and (2) across the entire study it was also calculated as the proportion of subjects achieving ≥ 150 min PA/wk at test time using the AAS for between-group comparisons.

## Results

Of the 692 subjects who underwent pre-exercise screening there were 27 (3.9%) at stage 1 and a further 32 (4.6%) at stage 2 who were recommended to seek medical clearance. Medical clearance was given for 57 of 59 subjects. Further medical follow-up was required for the remaining 2 subjects and exercise was contraindicated.

The subject characteristics for those starting the program are shown in Table 2. There was a small but significant difference in age distribution among the groups at the start of the study. No differences were found for gender proportions. The difference in weighted PA between the intervention subjects and active controls was approximately 10-fold at pre-intervention reflecting the high levels of PA for these regularly active subjects.

**Table 2:** Subject details pre-intervention for the three arms of the study.

	Group	Pedometer	Active Controls
Subjects [n]	148	251	135
Age at enrolment mean [± SD] yr	36.6 [± 12.5]*	40.1 [± 12.6]	39.1 [± 12.0]
Males n [%]	42 [28.4%]	57 [22.7%]	39 [28.9%]
Total PA [min/wk] weighted median [mean ± SD]	60 [71 ± 46]	70 [71 ± 47]	600 [706 ± 439]#
Total PA [min/wk] unweighted median [mean ± SD]	60 [65 ± 42]	60 [65 ± 43]	405 [477 ± 270]#

PA = physical activity; weighted PA calculated by multiplying vigorous-intensity minutes × 2 [24]

SD = standard deviation

* = significantly lower than pedometer subjects (p < 0.05)

# = significantly greater than intervention subjects (p < 0.05).

### Intervention compliance

**Figure 3:**
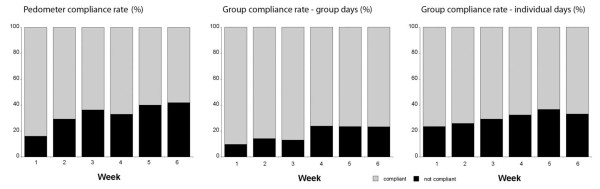
**Compliance rate for the pedometer subjects (% achieving the average daily step count across each week of the intervention) and group subjects on both group exercise days and on the subjects' individual exercise days (% achieving ≥ 30 min recorded activity per day across each week of the intervention)**.

Daily compliance during the intervention was calculated from diaries for the pedometer subjects and from HR monitor files for the group subjects (Figure 3). Overall compliance for the pedometer subjects was 67.2% across the intervention period. Compliance decreased progressively across the six week intervention phase (p < 0.05). Compliance for the group subjects was 74.7%. The average compliance was higher during the group sessions at 82.5% versus 70.0% for the individual days (p = 0.004) although both decreased across the six week intervention (p < 0.05). These ITT figures include days lost when subjects withdrew or did not use their HR monitor (even though they may have recorded a session in their diary) or pedometer.

### Long-term adherence and compliance

**Figure 4:**
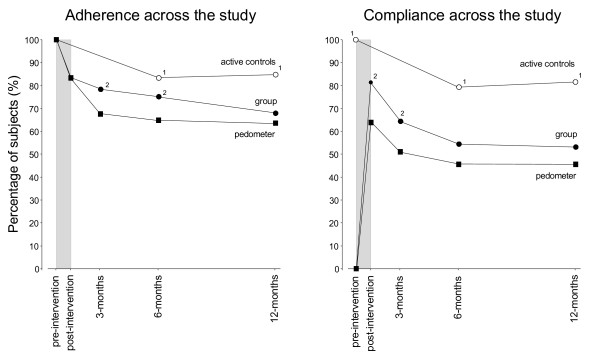
**The percentage of subjects remaining in the study (adherence) and achieving ≥ 150 min PA/wk (compliance) at the various test times**. Shaded area indicates the 40-DAY PA intervention phase. 1 indicates a higher rate between the active controls and intervention subjects. 2 indicates a higher rate for the group subjects versus the pedometer subjects using Chi square analysis (p < 0.05). Compliance was calculated using ITT analysis.

Figure 4 illustrates the patterns of adherence and compliance across the 12-month study. A significantly higher adherence pattern was found for the group versus pedometer subjects up to six months. Compliance (≥ 150 min PA/wk using the AAS) for the intervention subjects was highest post-intervention and then progressively decreased across the 12-month follow-up period. The group subjects showed greater compliance post-intervention and at 3-month follow-up relative to the pedometer subjects. The active controls maintained the highest adherence and compliance rates across the entire study.

**Figure 5:**
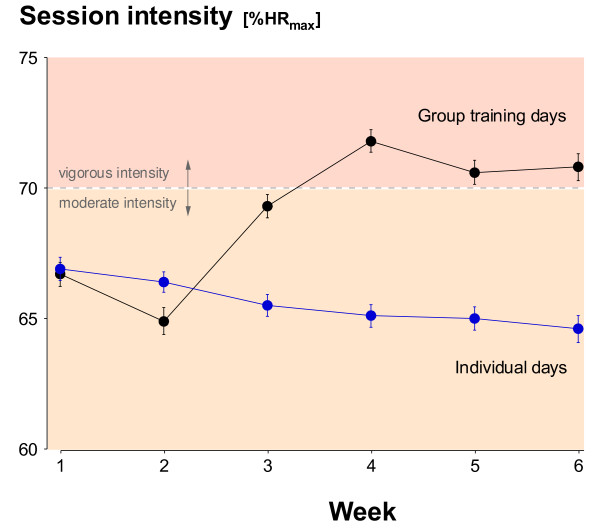
**The mean (± SE) intensity of the PA sessions by the group subjects**. Individual training days and group-led training days are shown separately.

The average exercise intensities for group sessions and individual days are shown in Figure 5. There was a progressive increase in intensity of the group classes (p < 0.0001) and a decreased intensity on the individual days (p = 0.017) across the intervention.

**Figure 6:**
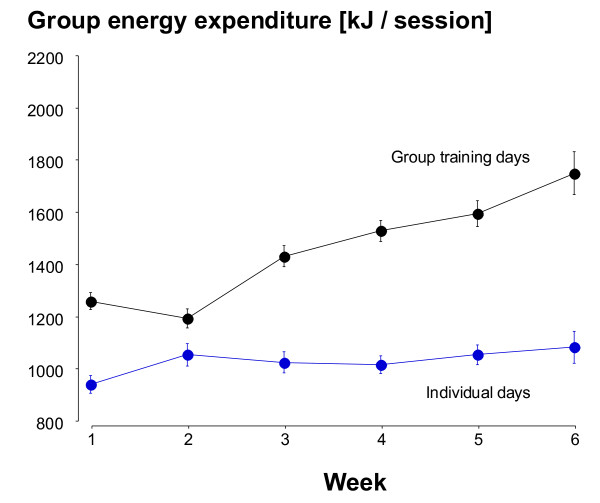
**The mean (± SE) kJ recorded during each PA session and averaged for each week of the intervention**. Individual training days and group-led training days are shown separately.

Figure 6 shows measured EE in the group sessions increased across the intervention (p < 0.0001) while it was unchanged in the individual sessions.

During the ~15,300 person-hr of activity for the intervention subjects there were 13 reports of musculo-skeletal injuries resulting in withdrawal from the project during the intervention phase. There were no adverse cardiovascular, metabolic or respiratory events despite a significant increase in vigorous PA reported by 79% of the intervention subjects (mostly group subjects) at the post-intervention testing. No difference in the rate of injury was found between the two intervention arms.

## Discussion

The intervention arms of the study utilised two different theoretical approaches, both designed to increase PA levels of insufficiently active adults. The results showed significant differences in the patterns of PA during and following the intervention phases of the study.

The first theoretical approach was a health information-based pedometer program.

The second approach was a group intervention. This involved a layered PA promotion strategy broadly based on the socio-ecological model of behaviour change. There were several common features of the two intervention arms (including those listed above) although there were some key differences as outlined in Table 1.

The different PA patterns between the more-layered group approach and the 'usual-treatment' pedometer program were significant to 6-months for adherence and 3-months for compliance (≥ 150 min PA/wk). In other words, there were intervention-specific characteristics that led to more subjects remaining in the program for longer and to a greater likelihood of those subjects reaching PA levels considered sufficient for health benefits both during and after the intervention. There were numerous additional strategies superimposed on the group intervention relative to the pedometer arm making it difficult to pin-point any one element leading to the increased group adherence and compliance. What is consistent is that group interventions have previously been found to result in better adherence than home-based or individual programs [39]. Exercising with others involves peer support and group cohesion and these are likely to play important roles in maintaining activity levels [40,41]. This is supported by both the differences between the intervention arms and between the group and individual days for the group-based subjects (Figures 3, 5 and 6). The additional residual effects of the group-based intervention lasted beyond the intervention itself although it eventually disappeared beyond 6-months. Sustainability for the group subjects was not designed around providing group sessions continuously but rather providing them early in the process of experiencing new activities and increasing self-efficacy [42].

A 40-day intervention duration was chosen to allow time for physiological improvements so participants could experience first hand new levels of fitness and energy. This has also been shown to be a critical time when those new to PA often undertake exercise inappropriately, become sore or disillusioned through unrealistic expectations or are concerned and confused about how to be active [36]. Following the intervention there was complete withdrawal of contact from all subjects except to re-schedule follow-up health and fitness checks. This was a design of the program to determine the impact and residual of the intervention arms.

Initial PA levels for the intervention subjects were low with a median of 71 min of weighted PA per week. In both intervention arms the proportion of subjects reaching and maintaining sufficient levels of PA was significantly higher than pre-intervention and remained this way throughout the 12-month follow-up (46.6% and 53.5% sufficiently active for the pedometer and group subjects, respectively at 12-month follow-up). This highlights the successes that can be achieved even with relatively low initial interaction among the pedometer subjects. Interestingly, there was a drop in the proportion of active controls who maintained sufficient levels of PA across the 12-month follow-up. Communication with the non-adherers indicated several were injured, one was pregnant, six had moved away and others were unwilling or unable to continue. Among those returning for testing about 5% of 'regular' exercisers fluctuated above and below recommended levels of activity. Nonetheless, the active controls as a group were clearly able to maintain very high levels of activity and proved stable as a reference group for health benefits.

It has been suggested that PA intervention studies should report elements such as injury rates among participants [8]. These data are often not collected or reported making it difficult to assess potential barriers and costs associated with increasing PA. In this study we have taken 399 insufficiently active adults from a low base and increased their PA over a relatively short period. Many subjects in both intervention arms undertook vigorous activity yet injuries during the interventions were relatively low. This is reassuring in that gradual exercise prescription for adults with low activity levels and risk factors can be safely achieved.

Finally, subjects from all groups commented on the reinforcing value of the periodic health and fitness checks which may have played a role in encouraging adherence among subjects from all groups.

### Limitations

The subjects ranged widely from students, health professionals, academics, public servants, cleaners etc; mapping of residency codes indicated a predominance of subjects living in the top 40% of the most advantaged metropolitan areas [43]. This factor is likely to be an important element in the relative success of the program although all intervention subjects began with a risk factor for low PA. The use of a self-reported questionnaire to measure PA levels is open to social desirability bias however improved health and fitness parameters (reported in another paper) confirmed substantial behaviour changes. The different types of exercise monitoring equipment between the two intervention arms may have different motivational influences on PA behaviour. However, it would appear that the use of equipment *per se *had less influence on PA behaviour as when the same equipment was used among the group subjects there were clear differences in PA behaviours between the group-led and individual sessions.

## Conclusions

Most authors recognise the importance of robust research to add clarity to the evidence-base for PA interventions that can effect behavioural change. The large cohorts of insufficiently active adults in both short-term interventions showed significant increases in PA patterns to 12-months post intervention. The subjects exposed to a range of elements within the group-based intervention demonstrated significantly higher adherence and compliance rates relative to the pedometer program following the intervention phase.

Getting people to change their behaviour, and to sustain healthy behaviours for extended periods of time, is always difficult. This paper details two types of exercise prescription strategies. Both show substantial longer-term results that would be significant for public health if translated to a population level.

## List of abbreviations

40-DAY PA: 40-day Physical Activity Study; AAS: Active Australia Survey; ANOVA: Analysis of variance; EE: Energy expenditure; HR: Heart rate; HRmax: Heart rate maximum; ITT: Intention to treat; PA: Physical activity.

## Competing interests

The authors declare that they have no competing interests.

## Authors' contributions

LHN & KIN conceived of the study and participated in the study design and coordination, collected the data, performed the statistical analysis and drafted the manuscript. NL participated in the study coordination, supervised the research group, participated in data acquisition and helped draft the manuscript. JD assisted in the study design. All authors read and approved the final manuscript.

## Authors' information

KIN is a professor of Exercise Science at the University of South Australia, he received an Australian Research Council linkage grant to undertake the 40-DAY PA study in conjunction with the South Australian Department of Health.

LHN is a lecturer in health promotion at Flinders University, the 40-DAY PA study was the principle intervention for her PhD.

NL was the project manager throughout the 40-DAY PA study

JD is a senior lecturer in exercise physiology at the University of South Australia.
